# Synergy of Liquid‐Crystalline Small‐Molecule and Polymeric Donors Delivers Uncommon Morphology Evolution and 16.6% Efficiency Organic Photovoltaics

**DOI:** 10.1002/advs.202000149

**Published:** 2020-06-18

**Authors:** Cenqi Yan, Hua Tang, Ruijie Ma, Ming Zhang, Tao Liu, Jie Lv, Jiaming Huang, YanKang Yang, Tongle Xu, Zhipeng Kan, He Yan, Feng Liu, Shirong Lu, Gang Li

**Affiliations:** ^1^ The Hong Kong Polytechnic University ShenZhen Research institute Shenzhen 518057 China; ^2^ Department of Electronic and Information Engineering The Hong Kong Polytechnic University Hung Hum Kowloon Hong Kong 999077 China; ^3^ Organic Semiconductor Research Center Chongqing Institute of Green and Intelligent Technology Chongqing 400714 China; ^4^ Department of Chemistry and Hong Kong Branch of Chinese National Engineering Research Center for Tissue Restoration & Reconstruction Hong Kong University of Science and Technology (HKUST) Clear Water Bay Kowloon Hong Kong 999077 China; ^5^ Department of Physics and Astronomy and Collaborative Innovation Center of IFSA (CICIFSA) Shanghai Jiaotong University Shanghai 200240 China

**Keywords:** liquid‐crystalline molecules, morphology, organic solar cells, ternary structures

## Abstract

Achieving an ideal morphology is an imperative avenue for enhancing key parameters toward high‐performing organic solar cells (OSCs). Among a myriad of morphological‐control methods, the strategy of incorporating a third component with structural similarity and crystallinity difference to construct ternary OSCs has emerged as an effective approach to regulate morphology. A nematic liquid‐crystalline benzodithiophene terthiophene rhodamine (BTR) molecule, which possesses the same alkylthio‐thienyl‐substituted benzo moiety but obviously stronger crystallinity compared to classical medium‐bandgap polymeric donor PM6, is employed as a third component to construct ternary OSCs based on a PM6:BTR:Y6 system. The doping of BTR (5 wt%) is found to be enough to improve the OSC morphology—significantly enhancing the crystallinity of the photoactive layer while slightly reducing the donor/acceptor phase separation scale simultaneously. Rarely is such a morphology evolution reported. It positively affects the electronic properties of the device—prolongs the carrier lifetime, shortens the photocurrent decay time, facilitates exciton dissociation, charge transport, and collection, and ultimately boosts the power conversion efficiency from 15.7% to 16.6%. This result demonstrates that the successful synergy of liquid‐crystalline small‐molecule and polymeric donors delicately adjusts the active‐layer morphology and refines device performance, which brings vibrancy to the OSC research field.

## Graphical abstract

The nematic liquid‐crystalline small‐molecule donor benzodithiophene terthiophene rhodamine (BTR) works as a successful third component in PM6:Y6‐based organic solar cells. The doping of BTR significantly enhances the crystallinity with slightly reducing donor/acceptor phase separation of the photoactive layer. This uncommon morphology evolution facilitates charge separation, transport, and collection and ultimately boosts the efficiency from 15.7% to 16.6%.

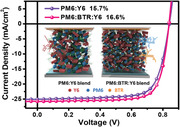

Organic solar cells (OSCs) are rising as a potential rival for delivering both high power conversion efficiencies (PCEs) and low large‐scale manufacturing costs, compared with the traditional silicon devices.^[^ ^1^, ^2^, ^3^, ^4^, ^5^, ^6^, ^7^, ^8^ ^]^ The last decade has witnessed the vitality of the OSC research field, which is brought by the concerted effort of materials engineering, device optimization, and device physics.^[^ ^9^, ^10^, ^11^, ^12^, ^13^, ^14^, ^15^, ^16^, ^17^, ^18^, ^19^, ^20^, ^21^, ^22^, ^23^, ^24^, ^25^, ^26^, ^27^, ^28^, ^29^, ^30^, ^31^, ^32^, ^33^, ^34^ ^]^

As organic semiconductors typically present low dielectric constant and short exciton diffusion length, the control of donor/acceptor (D/A) blend morphology, which is the integrated result of the nature of donors and acceptors (e.g., solubility, crystallinity, and miscibility), the film processing, and the device configuration, is crucial for achieving efficient charge separation, transport, and collection. A number of device‐engineering methods have been employed to regulate morphology, such as optimizing D/A ratio, opting solvents and additives, thermal annealing, solvent annealing, and post solvent treatment.^[^ ^35^, ^36^, ^37^, ^38^, ^39^ ^]^ Disappointingly, the research on morphological control is usually scattered and even fragmentary, and some working mechanisms are still unclear. Morphology in OSC is complicated and is almost always regulated by trial and error. Moreover, these conventional techniques always inevitably enlarge domain size excessively and sacrifice charge separation when crystallinity is evidently strengthened, which throws the morphological control work into a dilemma.^[^ ^40^ ^]^ To our best knowledge, very few works have reported the coexistence of obviously stronger crystallinity and reduced phase separation via device engineering.

Ternary OSCs have become an important research branch of device engineering, on account of their validity in overcoming the weaknesses of binary‐blend OSCs and retaining the simplicity of single‐junction OSCs.^[^ ^41^, ^42^, ^43^, ^44^, ^45^, ^46^, ^47^, ^48^ ^]^ The third component usually exerts one or multi functions of broadening/strengthening the absorption spectra, facilitating charge transfer/transport/collection, reducing carrier recombination, and optimizing the active‐layer morphology.^[^ ^45^, ^49^, ^50^, ^51^, ^52^, ^53^ ^]^ Despite the aforementioned challenges of achieving ideal morphology, the success of utilizing the third component of a similar structure with the host materials represents a key concept to regulate morphology.^[^ ^54^ ^]^ Early in 2015, by comparing successful and failed multiple‐donor systems, Yang and co‐workers found that the harmonious coexistence of two structural compatible polymeric donors (PTB7 and PBDTT‐SeDPP) endowed the PTB7:PBDTT‐SeDPP:PC71BM OSCs with a high PCE of 8.7%.^[^ ^55^ ^]^ Also, the cooperation of ITCPTC and MeIC, two structure‐similar and absorption‐similar small‐molecule acceptors helps fine‐tune the crystallinity and phase separation of the PM6:ITCPIC:MeIC blend. Besides, the crystallinity of the involved active‐layer materials is considered as another vital parameter to control morphology.^[^ ^56^ ^]^ The polymeric donor (Si‐PCPDTBT) of strong crystallinity demonstrates a good example.^[^ ^57^ ^]^ A set of Si‐PCPDTBT‐based ternary OSCs using P3HT:PC_61_BM, P3HT:ICBA, and PTB7: PC_71_BM host systems have been reported, where the introduction of Si‐PCPDTBT facilitates charge transport, suppresses charge recombination, and elevates PCE.^[^ ^58^, ^59^ ^]^ The concepts of structure similarity and crystallinity difference are combined in the recent all‐small‐molecule work of our group, where the incorporation of a dehydroxylated derivative of benzodithiophene terthiophene rhodamine (BTR), namely, BTR‐OH into the BTR:PC_71_BM system affords a champion PCE of 10.14% at a substantial blend thickness of 300 nm.^[^ ^60^ ^]^ It is because the less crystalline BTR‐OH weakened the crystallinity of the donor phase and optimized the D/A phase separation to a suitable scale, and thereby balanced the hole and electron transport. In spite of the effectiveness of structure‐similar but crystallinity‐different strategy, the application scope needs further extension.

BTR is a high‐performing small‐molecule donor material with excellent optoelectronic properties and nematic liquid crystal behavior. Hence, BTR presents strong intermolecular interaction, which is beneficial for maintaining a long‐range directional order and possessing high hole mobility.^[^ ^61^ ^]^ On the one hand, PM6 and BTR both comprise alkylthio‐thienyl‐substituted benzo building block, which presumably guarantees their good compatibility. On the other hand, the nematic liquid‐crystalline BTR has obviously stronger crystallinity than PM6.^[^ ^62^ ^]^ Last year, Zou group designed and synthesized a nonfullerene acceptor Y6, which features a ladder‐type electron‐deficient core‐based central fused ring (di‐thienothiophen[3.2‐*b*]‐pyrrolobenzothiadiazole) with a benzothiadiazole (BT) core. Outstanding PCEs of 15–16% are achieved by using wide bandgap polymer donors like PM6. The broad and high photoresponse was recorded at a high open‐circuit voltage, implying energy loss was as low as ≈0.5–0.6 eV. This superior performance of Y6 makes itself widely used in recent research work. Encouraged by the strategy of structure similarity and crystallinity difference, we incorporate BTR into the PM6:Y6 binary blend to further refine morphology and efficiency.^[^ ^63^ ^]^ It is worth mentioning that the doping of BTR (5 wt%) evidently enhances the crystallinity of the photoactive layer and slightly weakens the phase separation simultaneously, which is rarely reported in the literature. This morphology evolvement is associated with prolonged carrier lifetime, shortened photocurrent decay time, improved charge separation, transport, and collection, which ultimately boosts the PCE from 15.7% to 16.6%. This work elucidates the delicate effect of morphology tuning with the collaboration of liquid‐crystalline small‐molecule and polymeric donors and improves the OSC device performance.

**Scheme** **1**a,b displays the chemical structures and the energy levels of PM6, BTR, and Y6. BTR exhibits elevated highest occupied molecular orbital of −5.34 eV compared with PM6 (−5.56 eV), and they have similar lowest unoccupied molecular orbitals. The energy levels of PM6:Y6 and BTR:Y6 systems are well aligned to split photogenerated excitons. A set of OSCs are fabricated based on PM6:Y6, PM6:BTR:Y6, BTR:Y6 systems, with the conventional device structure of indium tin oxide (ITO)/poly(3,4‐ethylenedioxythiophene):poly(styrene sulfonate)(PEDOT:PSS)/active layer/PFNBr/Ag (Scheme 1c). 1‐chloronaphthalene (CN), a processing additive of high boiling point, is used to optimize the active‐layer morphology. As shown in **Figure** **1**a, PM6 and BTR display absorption onsets of 692 and 679 nm, indicative of their similar optical bandgaps of 1.79 and 1.83 eV, respectively. By comparison, the nonfullerene acceptor Y6 exhibits much red‐shifted absorption onset of 922 nm, illustrating its ultranarrow bandgap of 1.34 eV.

**Scheme 1:**
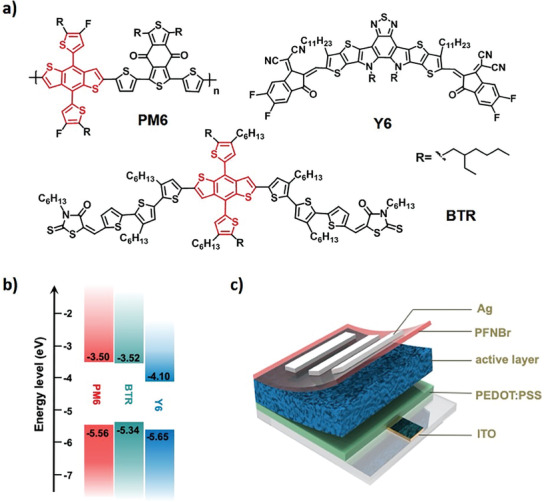
a) The chemical structures of PM6, BTR, and Y6. b) Energy level diagrams and c) the conventional device structure of ITO/PEDOT:PSS/active layer/PFNBr/Ag.

**Figure 1:**
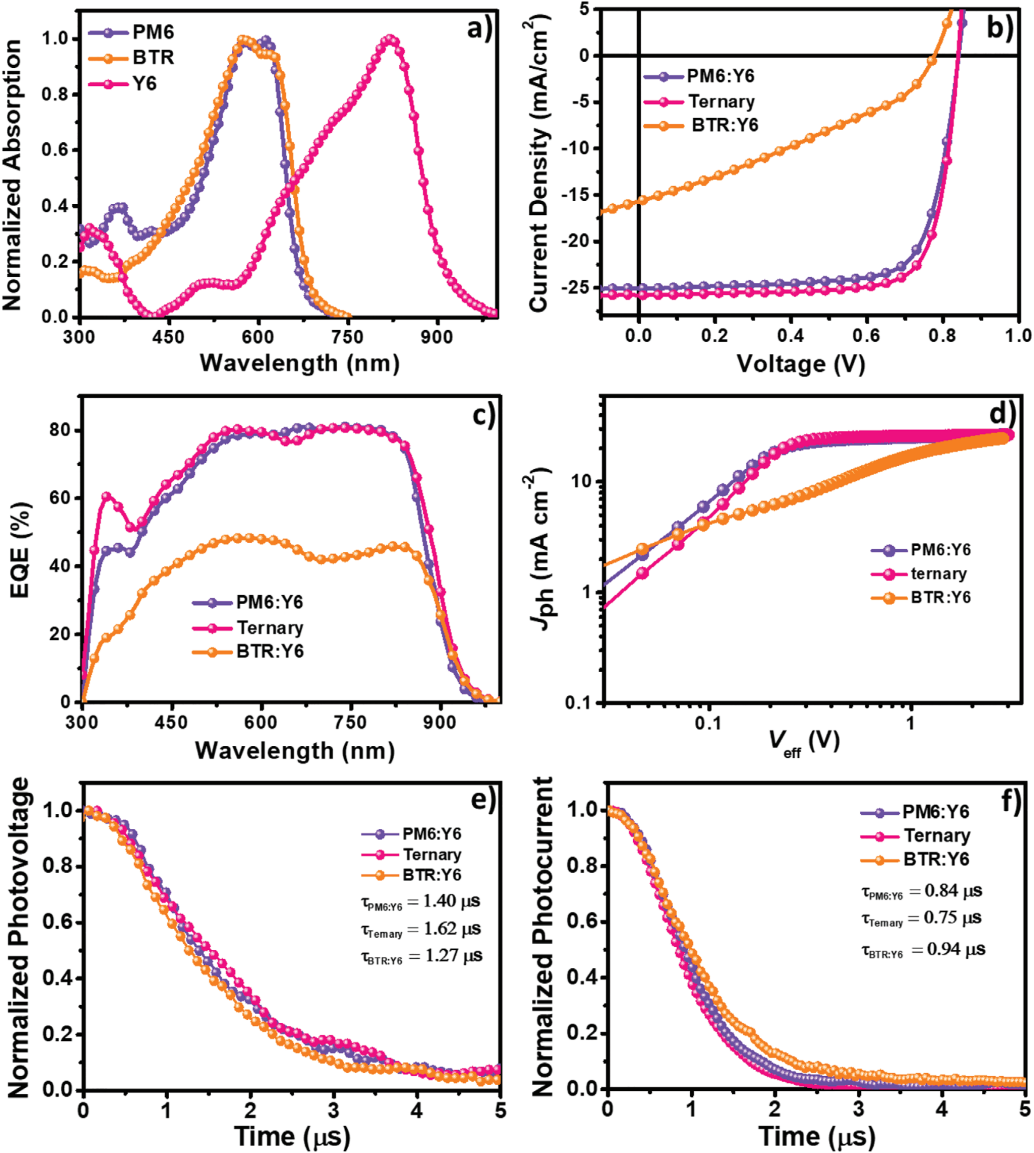
a) Normalized UV‐vis absorption of PM6, BTR, and Y6 pristine films. b) Current density–voltage (*J*–*V*) curves. c) EQE, d) *J*_ph_ versus *V*_eff,_ e) TPV, and f) TPC of OSC devices based on PM6:Y6, PM6:BTR:Y6 (0.95:0.05:1.2), and BTR:Y6 blends.

Figure 1b and Figure S1a (Supporting Information), and **Table** **1** and Table S1 (Supporting Information) summarize the current density–voltage (*J*–*V*) characteristics of optimized binary and ternary OSCs based on PM6:Y6, PM6:BTR:Y6, BTR:Y6 in different weight ratios. These OSCs are fabricated using the same process parameters for direct comparison. Binary device based on BTR:Y6 shows extremely low fill factor (FF) of 32.8% and PCE of 4.00%, with a short‐circuit current density (*J*_SC_) of 15.7 mA cm^−2^ and an open‐circuit voltage (*V*_OC_) of 0.776 V. In contrast, the PM6:Y6 blend exhibits a PCE of 15.7%, along with a *V*_OC_ of 0.840 V, a *J*_SC_ of 25.1 mA cm^−2^, and an FF of 74.4%, which is in accord with the literature. After adding 5 wt% loading of BTR into PM6:Y6 system, *J*_SC_ is effectively improved to 25.8 mA cm^−2^ and FF is obviously increased to 76.7%, which cover the negligible reduction of *V*_OC_ from 0.840 to 0.839 V and successfully elevate the efficiency to 16.6%. According to the external quantum efficiency (EQE) spectra, the respective integrated *J*_SC_’S of devices based on PM6:Y6, PM6:BTR:Y6, and BTR:Y6 blends are 24.5, 25.2, and 14.8 mA cm^−2^, which agree well with the *J*_SC_ value from the *J*–*V* curve (**Figure** **1**c).

**Table 1:** Photovoltaic parameters of PM6:BTR:Y6 device with weight ratios of 1:0:1.2, 0.95:0.05:1.2, and 0:1:1.2, with the structure of ITO/PEDOT:PSS /active layer/PFNBr/Ag, under simulated AM 1.5G irradiation at 100 mW cm^−2^

PM6:BTR:Y6	*V*_OC_ ^a)^ [V]	*J*_SC_ ^a)^ [mA cm^−2^]	Calc. *J*_SC_ ^b)^ [mA cm^−2^]	FF^a)^ [%]	PCE^a)^ [%]
1:0:1.2	0.840 (0.836 ± 0.005)	25.1 (25.0 ± 0.4)	24.4	74.4 (73.7 ± 1.0)	15.7 (15.4 ± 0.3)
0.95:0.05:1.2	0.839 (0.832 ± 0.007)	25.8 (25.7 ± 0.3)	25.2	76.7 (75.3 ± 1.1)	16.6 (16.1 ± 0.3)
0:1:1.2	0.776 (0.775 ± 0.006)	15.7 (15.7 ± 0.2)	14.8	32.8 (32.7 ± 0.8)	4.00 (3.97 ± 0.20)

a) Values for the highest PCE device, with average values obtained from 20 devices listed in parentheses;

b) *J*_SC_ value from the integration of the EQE spectra.

**Figure 2:**
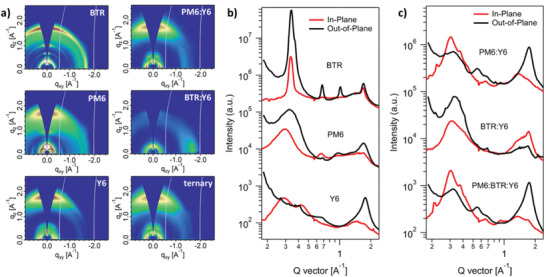
a) 2D GIXD patterns of neat and blend films. b) 1D line‐cuts of PM6, BTR, and Y6 neat films, and c) 1D line‐cuts of PM6:Y6, PM6:BTR:Y6 (0.95:0.05:1.2), and BTR:Y6 blend films.

To decipher the role of BTR in the PM6:BTR:Y6 ternary blend, charge separation and extraction properties are further studied by plotting the photocurrent density (*J*_ph_) as a function of the effective voltage (*V*_eff_) (Figure 1d and Table S2, Supporting Information). The photocurrent density is defined as *J*_ph_ = *J*_L_ − *J*_D_, where *J*_L_ and *J*_D_ refer to the current densities under illumination and in the dark, respectively. The effective voltage is defined as *V*_eff_ = *V*_0_ − *V*_A_, where *V*_0_ is the voltage when *J*_ph_ is equal to 0 and *V*_A_ is the applied bias voltage. It is assumed that, at a high *V*_eff_ of 3 V, the photogenerated excitons are fully dissociated into free holes and electrons, which are then totally collected by the respective electrodes. OSCs based on PM6:Y6 and PM6:BTR:Y6 blends presented a similar *J*_sat_ of ≈26.7 mA cm^−2^, evidently higher than BTR:Y6 devices (24.7 mA cm^−2^). This means the introduction of BTR has a negligible contribution to absorption. The exciton dissociation efficiency (*η*_diss_) and charge collection efficiency (*η*_coll_) are calculated quantitatively, according to the equations of *η*_diss_ = *J*_SC_/*J*_sat_ and *η*_coll_ = *J*_max_/*J*_sat_, respectively. The BTR:Y6 device displays extremely low *η*_diss_ of 63.6% and *η*_coll_ of 30.8%, which accounts for its disappointing performance. By contrast, the PM6:Y6 device reveals a *η*_diss_ of 93.9% and a *η*_coll_ of 83.8%, and the doping of BTR into PM6:Y6 blend availably improves *η*_diss_ and *η*_coll_ to 96.8% and 87.7%, indicative of the most efficient exciton dissociation and charge collection in the ternary blend.

In‐depth understanding of the charge recombination and extraction dynamics is attained from transient photovoltage (TPV) and transient photocurrent (TPC) measurements (Figure 1e,f). The carrier lifetimes (*τ*) of the open‐circuit conditions are extracted from the TPV decay dynamics under the irradiation of a simulated 1 sun using mono‐exponential fits. The ternary device displays a *τ* value of 1.62 µs, obviously larger than their parallels incorporating PM6:Y6 (1.40 µs) and BTR:Y6 (1.27 µs) blends, which unveils that charge recombination intensifies from PM6:BTR:Y6, PM6:Y6, to BTR:Y6 in order. TPC can probe the competition between carrier sweep‐out and recombination processes during OSC operation. The photocurrent decay time of the PM6:BTR:Y6 device under the short‐circuit condition is 0.75 µs, visibly shorter than that of the PM6:Y6 device (0.84 µs) and BTR:Y6 device (0.94 µs). It manifests that the combination of BTR and PM6 effectively facilitates charge extraction and accounts for the aforementioned highest charge collection efficiency of 87.7% for the ternary device.

The charge mobilities are fitted from the dark current density–voltage curves using the space‐charge‐limited current model (Figure S2 and Table S3, Supporting Information). Electron‐only devices are constructed with the architecture of ITO/ZnO/active layer/PFNBr/Al. The PM6:Y6 and BTR:Y6 blends display electron mobilities (*μ*_e_) of 2.96 × 10^−4^ and 2.05 × 10^−4^ cm^2^ V^−1^ s^−1^, which are far surpassed by the ternary‐blend rival with a *μ*_e_ of 3.59 × 10^−4^ cm^2^ V^−1^ s^−1^. The hole mobilities (*μ*_h_) are derived from hole‐only devices involving the ITO/MoO*_x_*/active layers/MoO*_x_*/Al structure. The BTR:Y6 blend exhibits a *μ*_h_ of 7.61 × 10^−4^ cm^2^ V^−1^ s^−1^, evidently higher than the PM6:Y6 parallel (4.77 × 10^−4^ cm^2^ V^−1^ s^−1^). The addition of BTR into PM6:Y6 blend effectively improves the *μ*_h_ to 5.53 × 10^−4^ cm^2^ V^−1^ s^−1^. The ratios of *μ*_h_ and *μ*_e_ are calculated to be 1.61, 1.51, and 3.71 in PM6:Y6, PM6:BTR:Y6, and BTR:Y6 blends, respectively. The highest and most balanced charge mobilities of PM6:BTR:Y6 blends account for its weakest charge recombination ascertained by TPV and its highest FF of 76.7%. By comparison, the extremely unbalanced mobilities of BTR:Y6 blends explain the strong charge recombination and the disappointing FF of 32.8% therein.

Grazing‐incidence X‐ray diffraction (GIXD) is employed to study the crystalline structure of neat and blend films. The 2D diffraction patterns and 1D line‐cuts are depicted in Figure 2, and the specific GIXD parameters are listed in Table S4 in the Supporting Information. For BTR neat film, the clear arc at *q* ≈ 1.70 Å^−1^ belongs to its *π*–*π* peaks, illustrating its 3D charge transport character. BTR film also displays sharp (100), (200), and (300) lamellar peaks at *q*_r_ ≈ 0.34, 0.66, 0.99 Å^−1^ along the in‐plane (ip) direction, and the (100) peak of BTR presents a large crystal coherence length (CCL) of 232.5 Å. The third‐order diffraction and the large CCL together evidence BTR's strong crystallinity. The PM6 neat film exhibits lamellar peaks and *π*–*π* peaks at *q*_z_ ≈ 0.295 and 1.70 Å^−1^ along the out‐of‐plane (oop) direction and thus demonstrates more amorphous nature than BTR. The NFA Y6 exhibits face‐on packing by displaying the oop *π*–*π* peaks at *q*_z_ ≈ 1.77 Å^−1^ and ip lamellar peaks at *q*_r_ ≈ 0.286 Å^−1^. As for the blend films, BTR:Y6 blend displays preferential edge‐on packing, which is unfavorable for charge transport toward electrodes. The PM6:Y6 and PM6:BTR:Y6 counterparts exhibit preferential face‐on packing by possessing oop (010) peaks at *q*_z_ ≈ 1.75 Å^−1^ (*d* ≈ 3.60 Å) and ip (100) peaks at *q*_r_ ≈ 0.30 Å^−1^ (*d* ≈ 20.9 Å). To carefully compare the crystallinity of blend films with and without BTR, the CCL and integrated area of (100) and (010) peaks are fitted. The PM6:BTR:Y6 blend displays respective CCLs of 97.8 and 22.9 Å for (100) and (010) peaks, slightly smaller than PM6:Y6 counterpart (98.5 and 24.1 Å). Despite the similarity of CCL, the ternary blend displays respective areas of 168.11 and 497.81(a. u.) for (100) and (010) peaks, more than twice as much as those of PM6:Y6 parallel (76.62 for (100) and 221.37 for (010)). **Scheme** **2** is the schematic of morphology evolution from PM6:Y6 to PM6:BTR:Y6 blend films, where the red, blue, and orange colors refer to Y6, PM6, and BTR, respectively. As it shows, the disparity of peak areas unveils that the BTR works as “nucleation agent” and endows the resulting ternary‐blend film with significantly stronger crystallinity, which in turn accounts for its enhanced *μ*_h_ and *μ*_e_, as well as the reduced charge recombination, in accordance with the TPV results.

**Scheme 2:**
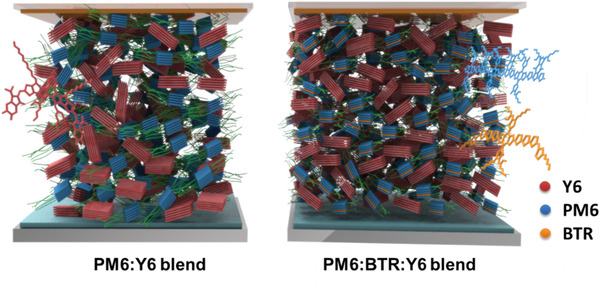
The schematic of morphology evolution from PM6:Y6 blend to PM6:BTR:Y6 blend.

Another important parameter of blend morphology, the donor/acceptor phase separation, is studied by atomic force microscopy (AFM) and transmission electron microscopy (TEM). As the AFM height images show (**Figure 3**a–c), the PM6, BTR, and Y6 pristine films have root mean square roughness (Rq) values of 1.05, 3.44, and 1.30 nm; and the PM6:Y6, PM6:BTR:Y6, and BTR:Y6 blends exhibit Rq of 1.51, 1.06, and 0.859 nm, respectively. As the AFM height images and TEM images show (Figure 3 and Figure S4, Supporting Information), PM6:BTR:Y6 blend displays smaller and more uniform phase separation than PM6:Y6 blends, which aids exciton dissociation. The BTR:Y6 blend film presents excessively large phase separation, which leads to the inadequate D/A interface area and thus severely restricts exciton dissociation. Note, the dramatic enhancement of crystallinity and the proper reduction of domain size, via the incorporation of BTR, is seldomly reported. In the literature, the significant enhancement of crystallinity typically excessively aggravates D/A phase separation, which impedes the win‐win situation of charge separation and transport.^[^ ^40^ ^]^

**Figure 3:**
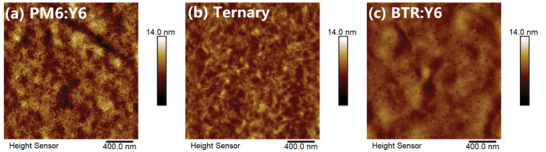
AFM height images of a) PM6:Y6, b) PM6:BTR:Y6 (0.95:0.05:1.2), and c) BTR:Y6 blend films.

In summary, we successfully designed an interesting ternary OSC based on harmonious coexistence of structural compatible nematic liquid‐crystalline small‐molecule donor (BTR) and polymeric donor (PM6) paired with NFA (Y6) that delivered a champion PCE of 16.6%, exceeding the PM6:Y6 and BTR:Y6 binary devices (15.7% and 4.0%). The role of BTR as a third component is systematically deciphered. We observed the simultaneous improvement of *J*sc, FF, and PCE, along with the enhanced charge separation, transport, and recombination. We also noticed the incorporation of BTR prolonged carrier lifetime from 1.40 to 1.62 µs, shortened photocurrent decay time from 0.84 to 0.75 µs, aided exciton dissociation efficiency from 93.9% to 96.8%, and enhanced charge collection efficiency from 83.8% to 87.7%. These improvements are primarily ascribed to the very unusual morphology evolution—the significantly enhanced crystallinity along with slightly reduced phase‐separation scale of the photo‐active active layer. The role of BTR, “nucleation agent,” is of particular importance when the device fabrication way is changed from spin‐coating fabrication to other large‐area fabrication ways (e.g., roll to roll, and blade coating). Such a strategy manifests the successful synergy of liquid‐crystalline small‐molecule donor and polymeric donor, which provides an alternative guideline to further fine‐tune the morphology toward new breakthroughs in OSCs.

## Conflict of Interest

The authors declare no conflict of interest.

## Supporting information

Supporting Information
