# Supplementary material for: Synergy of Liquid‐Crystalline Small‐Molecule and Polymeric Donors Delivers Uncommon Morphology Evolution and 16.6% Efficiency Organic Photovoltaics
Source: Adv Sci (Weinh). 2020 Jun 18;7(15):2000149. doi: 10.1002/advs.202000149 (PMC7404173; doi:10.1002/advs.202000149)
Supplement: Supplementary file 1 — Supporting Information [file ADVS-7-2000149-s001.pdf]

© 2020 Wiley-VCH GmbH

ADVANCED SCIENCE Open Access

Supporting Information

for *Adv. Sci.*, DOI: 10.1002/advs.202000149

Synergy of Liquid-Crystalline Small-Molecule and Polymeric Donors Delivers Uncommon Morphology Evolution and 16.6% Efficiency Organic Photovoltaics

Cenqi Yan, Hua Tang, Ruijie Ma, Ming Zhang, Tao Liu, Jie Lv, Jiaming Huang, YanKang Yang, Tongle Xu, Zhipeng Kan, He Yan, Feng Liu,* Shirong Lu, and Gang Li**

Electronic Supporting Information for

Synergy of liquid-crystalline small-molecule and polymeric donors delivers uncommon morphology evolution and 16.6%-efficiency organic photovoltaics

Cenqi Yan, Hua Tang, Ruijie Ma, Ming Zhang, Tao Liu, Jie Lv, Jiaming Huang, YanKang Yang, Tongle Xu, Zhipeng Kan, He Yan, Feng Liu*, Shirong Lu*, and Gang Li**

Dr. Cenqi Yan, Hua Tang, Jiaming Huang, Prof. Gang Li,

The Hong Kong Polytechnic University ShenZhen Research institute, Shenzhen 518057, China. E-mail: gang.w.li@polyu.edu.hk

Dr. Cenqi Yan, Hua Tang, Jiaming Huang, Prof. Gang Li,

Department of Electronic and Information Engineering, The Hong Kong Polytechnic University, Hung Hum, Kowloon, Hong Kong, China.

Ruijie Ma, Dr. Tao Liu, Prof. He Yan,

Department of Chemistry and Hong Kong Branch of Chinese National Engineering Research Center for Tissue Restoration & Reconstruction, Hong Kong University of Science and Technology (HKUST), Clear Water Bay, Kowloon, Hong Kong, China.

Hua Tang, Jie Lv, Tongle Xu, Prof. Zhipeng Kan, Prof. Shirong Lu

Chongqing Institute of Green and Intelligent Technology, Chongqing 400714, China, E-mail: lushirong@cigit.ac.cn

Ming Zhang, Dr. YanKang Yang, Prof. Feng Liu

Department of Physics and Astronomy and Collaborative Innovation Center of IFSA (CICIFSA), Shanghai Jiaotong University, Shanghai 200240, China, E-mail: fengliu82@sjtu.edu.cn

General information

All solvents and reagents were used as received from commercial sources and used without further purification. PBDB-T, BTR and Y6 were purchased from commercial source.

Morphology characterization

AFM measurements were obtained by using a Dimension Icon AFM (Bruker) in a tapping mode. Grazing incidence X-ray diffraction (GIXD) characterization of the thin films was performed at the Advanced Light Source on beamline 7.3.3, Lawrence Berkeley National Lab (LBNL). Thin film samples were spin-casted on to Si wafers. Si wafers were cleaned by sonication in detergent, deionized water, acetone and isopropyl alcohol. After that, the BHJ composite films were prepared on Si substrates using the same method as that for solar cell device fabrication. The scattering signal was recorded on a 2D detector (Pilatus 2M) with a pixel size of 0.172 mm by 0.172 mm. The samples were ≈15 mm long in the direction of the beam path, and the detector was located at a distance of ≈300 mm from the sample center (distance calibrated using a silver behenate standard). The incidence angle of 0.16° was chosen which gave the optimized signal-to-background ratio. The beam energy was 10 keV, operating at top-off mode. Typically, 30 s exposure time was used to collect diffraction signals. All GIXD experiments were done in helium atmosphere. The data was processed and analyzed using Nika software package.

Solar cell fabrication and characterization

Solar cells were fabricated in a conventional device configuration of ITO/PEDOT:PSS/active layers/PFNBr/Ag. The ITO substrates were first scrubbed by detergent and then sonicated with deionized water, acetone and isopropanol subsequently, and dried overnight in an oven. The glass substrates were treated by UV-Ozone for 30 min before use. PEDOT:PSS (Heraeus Clevios P VP AI 4083) was spin-cast onto the ITO substrates at 4000 rpm for 30 s, and then dried at 150 °C for 15 min in air. The donor/acceptor blends were dissolved in chloroform (the total concentration of blend solutions were 16 mg mL^{-1} for all blends) with 0.5 % 1-CN, and stirred overnight on a hotplate at 40°C in a nitrogen-filled glove box. The blend solution was spin-cast at 3000 rpm for 30 s. A thin PFNBr layer (0.5 mg mL^{-1} in methanol, 3000rpm for 30 s, about 15 nm) was coated on the active layer, followed by the deposition of Ag (100 nm) (evaporated under 5×10^{-5} Pa through a shadow mask). The optimal active layer thickness measured by a Bruker Dektak XT stylus profilometer was about 100 nm. The current density-voltage (J-V) curves of

devices were measured using a Keithley 2400 Source Meter in glove box under AM 1.5G (100 mW cm-2) using a Enlitech solar simulator. A 2×2 cm^2 monocrystalline silicon reference cell (SRC-1000-TC-QZ) was purchased from VLSI Standards Inc. The EQE spectra were measured using a Solar Cell Spectral Response Measurement System QE-R3011 (Enlitech Co., Ltd.). The light intensity at each wavelength was calibrated using a standard single crystal Si photovoltaic cell.

SCLC measurements

The electron and hole mobility were measured by using the method of space-charge limited current (SCLC) for electron-only devices with the structure of ITO/ZnO/active layer/PFNBr/Al and hole-only devices with the structure of ITO/MoO_x/active layers/MoO_x/Al. The charge carrier mobility was determined by fitting the dark current to the model of a single carrier SCLC according to the equation: $J = 9\varepsilon_0\varepsilon_r\mu V^2/8d^3$, where J is the current density, d is the film thickness of the active layer, μ is the charge carrier mobility, ε_r is the relative dielectric constant of the transport medium, and ε_0 is the permittivity of free space. $V = V_{app} - V_{bi}$, where V_{app} is the applied voltage, V_{bi} is the offset voltage. The carrier mobility can be calculated from the slope of the $J^{1/2} \sim V$ curves.

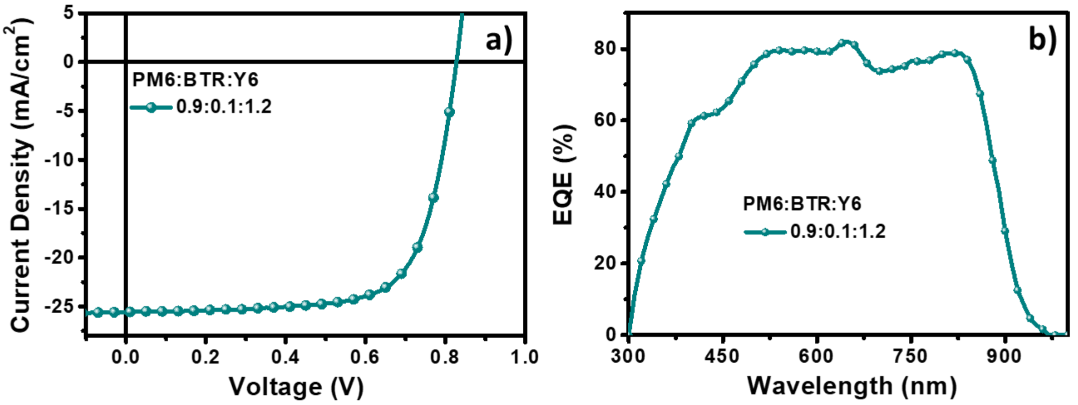

Figure S1 (a) Current density-voltage (*J-V*) curves and (b) EQE of OSC devices based on PM6:BTR:Y6 (0.9:0.1:1.2).

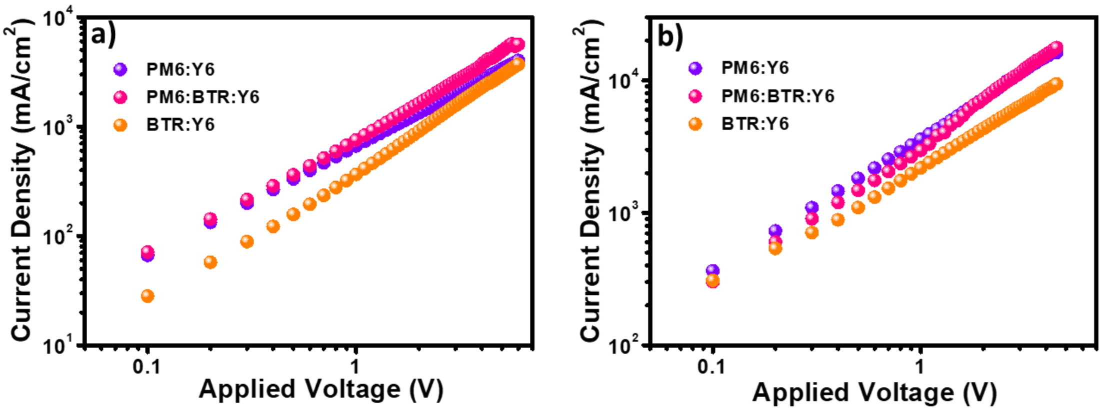

Figure S2 *J*-*V* characteristics in dark for (a) hole-only and (b) electron-only devices based on PM6:Y6, PM6:BTR:Y6 (0.95:0.05:1.2) and BTR:Y6 blends.

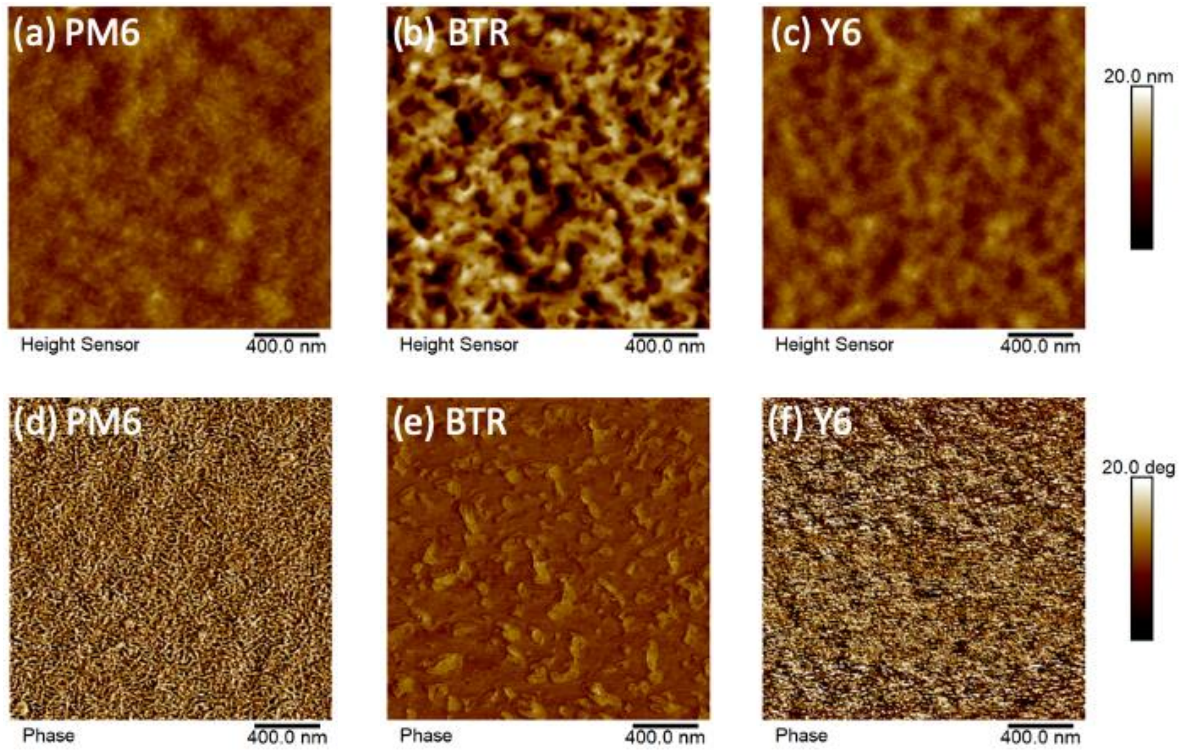

Figure S3 Atomic force microscopy (AFM) height (a-c) and phase (d-f) images of PM6, BTR and Y6 neat films.

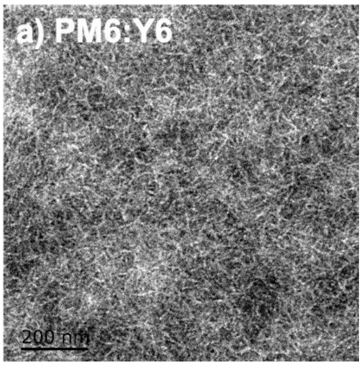

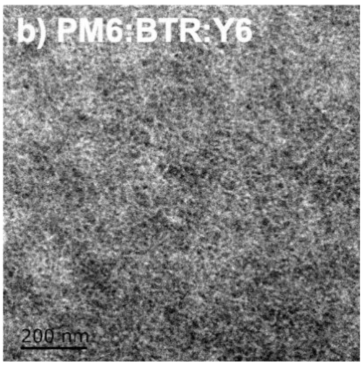

Figure S4 TEM images of (a) PM6: Y6 and (b) PM6:BTR:Y6.

Table S1 Photovoltaic parameters of PM6:BTR:Y6 device with weight ratios of 0.9:0.1:1.2, with the structure of ITO /PEDOT: PSS /active layer/PFNBr/Al, under simulated AM 1.5G irradiation at 100 mW cm^{-2}.

PM6:BTR:Y6	V_{OC} [a)] (V)	J_{SC} [a)] (mA cm^{-2})	calc. J_{SC} [b)] (mA cm^{-2})	FF [a)] (%)	PCE[a)] (%)
0.9:0.1:1.2	0.827 (0.825±0.005)	25.6 (25.3±0.3)	24.7	71.2 (70.2±0.9)	15.1 (14.7±0.3)

Table S2 charge mobilities of PM6:Y6, PM6:BTR:Y6 (0.95:0.05:1.2) and BTR:Y6 blend films.

PM6:BTR:Y6	Hole Mobility ($\times 10^{-4}$ cm^2 V^{-1} s^{-1})	Electron mobility ($\times 10^{-4}$ cm^2 V^{-1} s^{-1})	Ratio of hole mobility to electron mobility
1:0:1.2	4.77	2.96	1.61
0.95:0.05:1.2	5.53	3.59	1.54
0:1:1.2	7.61	2.05	3.71

Table S3 Exciton dissociation efficiency and charge collection efficiency of PM6, BTR and Y6 neat films, and PM6:Y6, PM6:BTR:Y6 (0.95:0.05:1.2) and BTR:Y6 blend films.

PM6:BTR:Y6	J_{sat} (mA cm^{-2})	J_{sc} [a] (mA cm^{-2})	J_{max} [b] (mA cm^{-2})	exciton dissociation efficiency	charge collection efficiency
1:0:1.2	26.696	25.059	22.362	93.9%	83.8%
0.95:0.05:1.2	26.654	25.790	23.387	96.8%	87.7%
0:1:1.2	24.685	15.703	7.611	63.6%	30.8%

[a] J_{ph} at the short-circuit condition [b] J_{ph} at the max power point

Table S4 GIWAXS fitting data of PM6, BTR and Y6 neat films, and PM6:Y6, PM6:BTR:Y6 (0.95:0.05:1.2) and BTR:Y6 blend films.

Material	Peak	Position	FWHM	Area	D-spacing	CCL
BTR	100	0.33707	0.02432	51.389	18.64005	232.5123
BTR	010	1.7015	0.1559	43.535	3.692624	36.27133
PM6	100	0.2947	0.10927	93.499	21.31999	51.74979
PM6	010	1.7049	0.35317	137.6	3.68526	16.01127
Y6	100	0.28556	0.11957	81.348	22.00238	47.29196
Y6	010	1.7692	0.24123	170.84	3.551323	23.44111
PM6:Y6	100	0.30224	0.057416	76.615	20.78812	98.48648
PM6:Y6	010	1.7473	0.23462	221.37	3.595834	24.10153
BTR:Y6	100	0.33656	0.11231	357.4	18.66829	50.34903
BTR:Y6	010	1.7344	0.23605	75.08	3.622578	23.95552
PM6:BTR:Y6	100	0.30299	0.057827	168.11	20.73666	97.7865
PM6:BTR:Y6	010	1.747	0.24654	497.81	3.596451	22.93624
